# The impact of work environment on caring behavior among Chinese hospice nurses: the chain mediating effect of psychological capital and empathy

**DOI:** 10.3389/fpsyg.2024.1348269

**Published:** 2024-04-30

**Authors:** Tiantian Wang, Yunrong Li, Jie Chen, Aifeng Meng, Yeping Wang, Liuliu Zhang, Bing Wu, Bo Yang, Yun Zhao

**Affiliations:** ^1^Nursing Department, Nanjing Medical University Affiliated Cancer Hospital/Jiangsu Cancer Hospital, Nanjing, China; ^2^School of Nursing, Nanjing University of Chinese Medicine, Nanjing, China; ^3^Analgesics Department, Nanjing Medical University Affiliated Cancer Hospital/Jiangsu Cancer Hospital, Nanjing, China; ^4^Urology Department, Nanjing Medical University Affiliated Cancer Hospital/Jiangsu Cancer Hospital, Nanjing, China

**Keywords:** work environment, caring behavior, psychological capital, empathy, China, hospice nurses

## Abstract

**Introduction:**

The caring behavior of hospice nurses toward patients positively impacts their professional careers and significantly improves the quality of hospice services. A positive and supportive work environment may protect nurses against negative emotions that may affect the humanistic care they provide, and their job satisfaction. This study aimed to explore the impact of the nursing work environment on caring behavior. We also investigated the chain mediating effect of psychological capital and empathy on this relationship among Chinese hospice nurses.

**Methods:**

The Practice Environment Scale (PES), the Psychological Capital Questionnaire (PCQ), the Empathy Ability Scale for Hospice Nurses, and the Caring Behaviors Inventory (CBI) were used to survey 393 Chinese hospice nurses. SPSS 27.0 and Mplus 8.0 were used for statistical processing to analyze the mediating effects.

**Results:**

The nursing work environment positively predicted caring behavior. Furthermore, it was found that psychological capital and empathy jointly mediate the relationship between the nursing work environment and caring behavior.

**Conclusion:**

This study reveals how the nursing work environment affects the caring behavior of hospice nurses. Hospital managers need to provide hospice nurses with a favorable working environment from the perspective of positive psychology, continuously monitor their psychological state, improve their caring behavior, and provide references for developing intervention plans to promote the caring behavior of hospice nurses in the future.

## 1 Introduction

Hospice care in China commenced belatedly and is still at the preliminary stage. With the intensification of population aging, changes in family structure and disease spectrum, there is a great demand for hospice care in China. However, the current coverage rate for hospice care in China stands at a mere 10% (Zhang et al., 2023). Caring behavior is the essence and core of nursing. It manifests as humanistic care and an active emotion and attitude of caring for others. Professional healthcare workers demonstrate caring behavior by protecting, helping, and supporting patients, helping them achieve physical, mental, spiritual, and socio-cultural health (Watson, 1999). Nurses frequently and closely contact patients throughout medical care. They are considered the most critical group in raising public awareness and providing quality hospice care, and their caring behavior for end-of-life patients directly affects the quality of hospice care services (Lu et al., 2018). In addition, their positive caring behavior significantly accelerates patients’ recovery, improves satisfaction, and promotes nursing quality (Yau et al., 2019). Compared with nurses in regular departments, hospice nurses usually deal with patients at the end of life, who often require more care and attention. This not only requires nurses to have high professional skills, solid theoretical knowledge, and strong psychological qualities, but also requires them to have good empathy, continuously provide humanistic care to patients and their families, and meet their psychological and emotional needs beyond their physiology (Liu et al., 2023) Directly and indirectly, their caring behaviors affect the end-of-life experience of patients and their families in hospice care services (Lai et al., 2022). Therefore, the level of care provided by nurses is particularly important in hospice care. However, other studies suggested that the overall level of care among hospice nurses in China is relatively low and the negative caring behaviors of hospice nurses can lead to compassion fatigue, job burnout, and frequent conflicts between nurses and patients (Deng et al., 2019; Mottaghi et al., 2020). Therefore, studying the caring behavior of hospice nurses is vital to improving their negative emotions, maintaining their careers, promoting further development of hospice care, and building harmonious nurse–patient relationships.

The nursing work environment refers to organizational characteristics that promote or limit nursing professional practice. They include nurse-manager relationships, physician-nurse relationships, and nursing positions in hospitals. Hospice nurses work in a complex environment due to their long-term exposure to patient death and family sadness. The lack of effective coping behavior can lead to the accumulation of sad emotions. Meanwhile, the nursing work environment can directly affect the nursing process and the safety of patients. Moreover, it can indirectly affect nurses’ job satisfaction, mental health, and occupational fatigue, affecting nursing quality (Wei et al., 2018) According to a study on clinical nurses, a healthy nursing work environment improves nurses’ emotional intelligence, making it easier to coordinate interpersonal relationships (Maillet and Read, 2021). Research confirms that nurses’ work environment is closely correlated with their quality of life and the satisfaction of their patients (Baek et al., 2020). According to a previous study, the nursing work environment directly predicts the caring behaviors of nurses, which implies that the caring behaviors of nurses are affected by the quality of their work environment (Arsat et al., 2022). Thus, the following hypothesis is proposed:

*Hypothesis 1*: The nursing work environment significantly predicts caring behavior.

Psychological capital is a positive psychological state that individuals exhibit during their growth and development. It specifically manifests as (a) having confidence (self-efficacy) and being able to make necessary efforts to achieve success when facing challenging work; (b) positive attribution (optimism) toward current and future success; (c) persistence in adjusting the path to achieving goals when necessary to achieve success (hope); (d) being able to persevere, quickly recover and surpass (resilience) to achieve success when facing adversity and being troubled by problems (Ding et al., 2015). Although there is no uniform definition of psychological capital in the current research, most definitions revolve around the four dimensions of self-efficacy, hope, resilience, and optimism. Numerous studies have discovered a significant positive correlation between psychological capital and variables such as job performance and organizational commitment (Avey et al., 2010; Wang et al., 2023). Moreover, research on medical professionals shows that the nursing work environment is positively associated with psychological capital. A supportive nursing work environment can develop psychological capital (Pan et al., 2017). Furthermore, studies on undergraduate nursing students suggest that positive psychological capital and its related dimensions are significantly and positively correlated with caring behaviors, except for self-efficacy (Jiang et al., 2017). Therefore, this study attempts to investigate the following hypotheses:

*Hypothesis 2*: Psychological capital might mediate the relationship between the nursing work environment and caring behavior.

Empathy means putting others at the center, choosing their opinions, feeling their emotions, and then generating similar or consistent emotional experiences and behavioral reactions with others (Bas-Sarmiento et al., 2020). Nursing communication is built on empathy and the emotional relationships between nurses and patients. Hospice nurses must continuously provide empathy and care to patients, enduring physical and mental pressure and bearing the emotional labor of patients’ families, which can easily lead to empathy fatigue (Jung and Matthews, 2021). Meanwhile, researchers demonstrated that a good environment intrinsically promotes the quality of nursing care. Nurses can also feel happiness and satisfaction while working, which reduces emotional exhaustion and improves empathy (Liu et al., 2014). Furthermore, empathy is positively correlated with caring behavior (Lina et al., 2022). When nurses have empathy, they experience their psychological processes from the patient’s perspective, accurately assess their emotional needs, and provide humanistic care. Therefore, we propose the following assumption:

*Hypothesis 3*: The relationship between the nursing work environment and caring behavior can be mediated by empathy.

Numerous studies reported that nursing work environment, psychological capital, and empathy are positively associated with caring behavior and job satisfaction among nurses (Kim and Lee, 1653; Hao et al., 2020). Researchers pointed out that psychological capital predicts empathy in Chinese nurses, which improves problem-solving ability and work engagement (Gao et al., 2023). In addition, the Triadic Reciprocal Determinism (Albert, 2017) suggests that environmental factors, individual factors, and individual behavior are interconnected and mutually influenced, intending to explain how an individual’s psychology and behavior are influenced by the interaction between environmental and individual factors. According to this theory, the interaction between individuals and the environment affects not only their behavior, but also their psychological state and cognitive processes. This theory can help understand the relationship between the nursing work environment (environment), psychological capital (individual factors), empathy (individual factors), and caring behavior (behavior) of hospice nurses. Consequently, we propose the following assumption:

*Hypothesis 4*: Psychological capital and empathy jointly mediate the relationship between the nursing work environment and caring behavior.

Although previous studies have shown a significant correlation between the nursing work environment and caring behavior, the existing literature still needs to be clarified on how the nursing work environment affects caring behavior through psychological capital and empathy. In addition, few studies have been conducted on hospice care; thus, this relationship among hospice nurses remains to be known. This study aimed to establish a model for exploring possible internal mechanisms between these four variables in hospice nurses and confirm psychological capital and empathy as mediators.

### 1.1 Hypothetical research model

**Figure 1:**
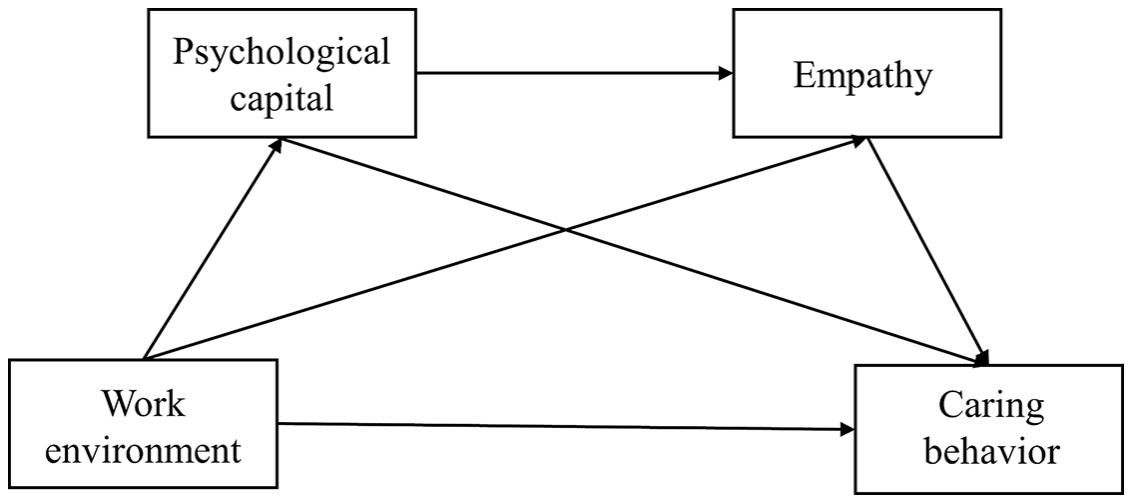
Hypothetical chain mediation model.

In this study, we focused on the impact of work environment on caring behavior among Chinese nurses. Moreover, we explored the potential mechanism of this connection with psychological capital and empathy as mediators. Our study formulated the below hypotheses (see Figure 1).

## 2 Materials and methods

### 2.1 Participants

This cross-sectional survey was conducted in September 2023. The study adopted a convenience sampling method and recruited 420 hospice nurses from different hospitals in China. They completed the survey through online questionnaires. Among 420 nurses, 393 were valid (the remaining participants were excluded because of missing data fields), and the effective response rate reached 93.57%. The inclusion and exclusion criteria were listed in Table 1. Table 2 shows the basic characteristics of the participants.

**Table 1:** The inclusion and exclusion criteria for hospice nurses.

The inclusion and exclusion criteria	Entries
Inclusion criteria	(a) Having a professional qualification certificate in China
	(b) At least 1-year working experience in palliative units
	(c) Being older than 18 years old
	(d) Having basic phone or computer skills
	(e) Providing informed consent and voluntary participation
Exclusion criteria	(a) Took a leave of absence longer than six months before the study
	(b) Informal employees, such as interns, visiting scholars, etc.
	(c) Failed to complete the survey

**Table 2:** Baseline characteristics and differences in the caring behavior score of nurses.

Variables	Frequency (percentage)	Caring behavior (M ± SD)	*t*/*F*	*P*
Gender			1.083	0.280
Male	4 (1%)	115.50 ± 21.56		
Female	389 (99%)	125.59 ± 18.53		
Age (years)			2.700	0.046
≤ 25	64 (16.3%)	123.20 ± 18.91		
26–30	56 (14.2%)	126.91 ± 18.13		
31–40	183 (46.6%)	123.61 ± 19.83		
41–50	84 (21.4%)	129.79 ± 15.02		
≥ 51	6 (1.5%)	133.83 ± 13.92		
Marital status			8.913	< 0.001
Unmarried	93 (23.7%)			
Married	289 (73.5%)			
Divorce	11 (2.8%)			
Monthly income			1.851	0.137
≤ 3,000	12 (3.1%)	136.67 ± 10.27		
3,001–6,000	90 (22.9%)	124.40 ± 19.28		
6,001–9,000	165 (42%)	124.50 ± 19.36		
≥ 9,001	126 (32.1%)	126.51 ± 17.29		
Health			7.468	< 0.001
Excellent	69 (17.6%)	131.68 ± 16.83		
Good	179 (45.5%)	127.39 ± 16.87		
Average	138 (35.1%)	120.19 ± 19.87		
Poor	7 (1.8%)	120.43 ± 24.47		
Post			9.606	< 0.001
Nurse	256 (65.1%)	122.93 ± 19.65		
Head nurse	75 (19.1%)	129.24 ± 16.77		
Responsible group leader	62 (15.8%)	131.52 ± 13.26		
Title			4.290	0.006
Nurse	51 (13%)	125.02 ± 19.97		
Nurse practitioner	92 (23.4%)	121.97 ± 18.85		
Nurse–in–charge	187 (47.6%)	125.45 ± 19.04		
Associate senior nurse	63 (16%)	131.14 ± 13.98		
Education			0.737	0.479
Junior college	36 (9.2%)	128.14 ± 19.58		
Undergraduate	346 (88%)	125.08 ± 18.53		
Postgraduate	11 (2.8%)	129.73 ± 16.18		
Working years in hospice			1.048	0.382
1–3	241 (61.3%)	124.14 ± 18.95		
4–6	77 (19.6%)	128.09 ± 17.06		
7–10	36 (9.2)	129.08 ± 17.23		
11–15	26 (6.6%)	125.85 ± 20.64		
≥ 15	13 (3.3%)	124.54 ± 18.71		
Employment type			0.874	0.427
Contract	255 (64.9%)	124.91 ± 19.26		
Personnel agency	13 (3.3%)	121.54 ± 21.72		
Formal preparation	125 (31.8%)	127.10 ± 16.68		
Experience of training			2.907	0.004
Yes	306 (77.9%)	126.93 ± 17.62		
No	87 (22.1%)	120.44 ± 20.86		

### 2.2 Measures

All measures in this study were conducted in Chinese language.

### 2.3 Social-demographic characteristics

A self-made demographic questionnaire was utilized in this study to collect the characteristics of participants, including gender (male, female), age, educational background (college, bachelor’s, or graduate degree), work year, marital status (unmarried, married, divorced), health condition (bad, general, good, very good), professional title (junior, intermediate and above), job position, and monthly income.

### 2.4 Nursing work environment

The Practice Environment Scale (PES) was developed by Lake (2002) and revised in the Chinese version by Wang and Li (2011). The scale included 28 items which were divided into 5 dimensions, including “nurses participating in hospital affairs” (8 items), “foundation of high-quality nursing services” (9 items), “ability and leadership style of managers” (4 items), “sufficient human and material resources” (4 items), and “medical and nursing cooperation” (3 items). Participants were rated on a four-point Likert response format (from 1 = “completely disagree” to 4 = “completely agree”). The higher score indicated a better nursing work environment. For the current sample, Cronbach’s α was 0.976.

### 2.5 Psychological capital

The Psychological Capital Questionnaire (PCQ) was used to evaluate nurses’ psychological capital. It was designed by Luthans et al. (2004) and translated into Chinese by Luo and He (2010). The questionnaire consisted of 20 items, divided into four dimensions: self-efficacy, hope, resilience, and optimism. Each item was rated on a 6-point Likert scale, with 1 representing “strongly disagree” and 6 representing “strongly agree.” Cronbach’α coefficient for the PCQ was 0.980 in this study, with a higher score indicating better psychological capital.

### 2.6 Empathy

This study employed the Empathy Ability Scale for Hospice Nurses designed and compiled by Wang and Li (2023) The scale consisted of 3 dimensions with 33 items. The 3 dimensions included “cognitive empathy,” “emotional empathy,” and “behavioral empathy.” This scale assessed nurse self-evaluation, using the Likert 5-level scoring method. From “none” to “always,” 1–5 points were calculated, and the score of each dimension was equal to the total score of the dimension items. A higher dimension score indicated stronger empathy ability reflected by the dimension. The Cronbach’s α for this scale was 0.987 in this study.

### 2.7 Caring behavior

Wolf designed the Caring Behaviors Inventory (CBI) in Wolf et al. (1994). In 2006, Wu et al. reduced it to 24 items (Wu et al., 2006). This study used the Chinese version of the CBI scale, which Da Chaojin translated (Da et al., 2017). This scale had three dimensions, mainly support and assurance, knowledge and skills, respect and connection, with 24 items. Nurses agreed to 6 Likert responses from 1 to 6, with “1″ = “never” and “6″ = “always.” A higher total score indicated better caring behavior. In this study, the Cronbach’s α coefficient of this scale was 0.979.

### 2.8 Procedure

This study used professional online questionnaire software to generate questionnaire links, which were anonymously filled out. After obtaining the consent of hospital leaders, the questionnaire links were distributed to the nursing department directors through online platforms. They then organized nurses who met the inclusion criteria to complete the questionnaire. Before the investigation, participants studied the informed consent form and expressed their willingness to accept the survey before entering the formal answering interface. Before filling out the questionnaire, relevant information was explained, and precautions were taken. This was to ensure the accuracy of the answers. Nurses could complete the questionnaires through various devices such as computers, tablets, and mobile phones. To prevent duplication, the backend set up the same account and device to complete questionnaires only once. All data was securely stored. The Ethics Committee of the Jiangsu Cancer Hospital approved this study.

### 2.9 Statistical analysis

SPSS 27.0 and Mplus 8.0 were used for statistical analysis. The count data of baseline characteristics are presented as frequency and percentage [n (%)], while measurement data are presented as mean and standard deviation [M ± SD]. *T*-test or one-way ANOVA was adopted to analyze factors affecting the caring behavior of nurses. Thereafter, Pearson correlation analysis was used to determine the correlation between the nursing work environment, psychological capital, empathy, and caring behavior. The above processes were conducted using SPSS 27.0. In addition, Mplus 8.0 was used to analyze the mediating effect of psychological capital and empathy between the nursing work environment and caring behavior. The significance of the mediation effect was measured using Bootstrap with 5,000 repeated samples and a two-sided inspection level α = 0.05.

### 2.10 Control variables

According to the statistical analysis results in Table 2, current research takes the hospice nurses’ age, health status, position, nursing title, marital status, and training experience as the control variables.

## 3 Results

As shown in Table 2, 393 participants with an average age of 34.32 (SD = 7.4) were included in the final valid sample. 73.5% of participants fell into the married group, and most of them were in good or excellent health (63.1%). Approximately two-thirds held primary positions (65.1%). Concerning nurse title, almost half of the participants held intermediate titles (47.6%). A majority of participants reported receiving humanistic care training (77.9%).

### 3.1 Common method biases test

We used Harman’s single-factor test for all questions on the four scales before data processing. In this study, there are six factors with eigenvalues >1, and the variation explained by the first factor is 32.73%, which is less than the critical criteria of 40%, indicating that the common method bias is not apparent.

### 3.2 Level of caring behavior

The average value of the overall caring behaviors in our study was 125.5 ± 18.6. The average value of each dimension was as follows: support and guarantee 26.9 ± 3.8; knowledge and skills 28.4 ± 6.9; respect and connection 50.2 ± 9.0 (Table 3).

**Table 3:** Descriptive statistics of various variables.

Scale/Subscale	Mean	SD
1. Caring behavior	125.5	18.6
1.1 Support and guarantee	26.9	3.8
1.2 Knowledge and skills	28.4	6.9
1.3 Respect and connection	50.2	9.0
2. Work environment	90.5	13.6
3. Empathy	138.2	24.6
4. Psychological capital	101.4	14.7

### 3.3 Descriptive statistics

The average score on the work environment scale was 90.5 ± 13.6 (Table 3). As for empathy, the overall average value was 138.2 ± 24.6. The average psychological capital value among these participants was 101.4 ± 14.7.

### 3.4 Correlation analysis

According to the relationships between variables in Table 4, the nursing work environment was positively related to psychological capital (*r* = 0.768, *p* < 0.01), empathy (*r* = 0.678, *p* < 0.01), and caring behavior (*r* = 0.632, *p* < 0.01). Psychological capital was positively correlated with empathy (*r* = 0.706, *p* < 0.01) and caring behavior (*r* = 0.644, *p* < 0.01). Moreover, empathy was positively associated with caring behavior (*r* = 0.807, *p* < 0.01).

**Table 4:** Associations between the variables.

Variables	1	2	3	4
1. Caring behavior	1			
Work environment	0.632^**^	1		
3. Empathy	0.807^**^	0.678^**^	1	
4. Psychological capital	0.644^**^	0.768^**^	0.706^**^	1

**p < 0.01.

### 3.5 The mediation effect analysis

The total (*β* = 0.862, *t* = 15.7, *p* < 0.001) and direct (*β* = 0.151, *t* = 2.3, *p* < 0.05) effects of the nursing work environment on the caring behavior both remained significant after controlling the variables such as age, health status, position, nursing title, marital status, and training experience (Table 5). The nursing work environment significantly predicted psychological capital (*β* = 0.768, *t* = 35.2, *p* < 0.001) and empathy (*β* = 0.332, *t* = 4.9, *p* < 0.001). In addition, caring behavior was significantly predicted by empathy ability (*β* = 0.672, *t* = 14.9, *p* < 0.001).

**Table 5:** Regression analysis of the relationship between variables.

Outcome variables	Predictor variables	*β*	*T*	LLCI	ULCI	*p*
Caring behavior	Work environment	0.111	2.3	0.027	0.288	*p* = 0.022
	Empathy	0.672	14.9	0.431	0.576	*P* < 0.001
	Psychological capital	0.085	1.5	−0.030	0.242	*p* = 0.125
Empathy	Work environment	0.332	4.9	0.355	0.833	*p* < 0.001
	Psychological capital	0.451	6.6	0.525	0.990	*p* < 0.001
Psychological capital	Work environment	0.768	35.2	0.762	0.907	*p* < 0.001

**Figure 2:**
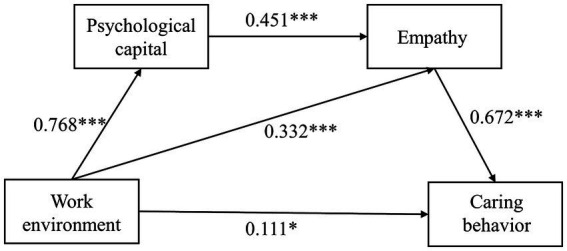
Model diagram of the effect of work environment on caring behavior (*p < 0.05, ***p < 0.001).

The Bootstrap method (5,000 repeated sampling) was used in the mediation effects test. Table 6 shows that the total indirect mediation effect did not contain 0 [Bootstrap 95% CI: 0.591, 0.836], accounting for 82.5% of the total mediation effect. Two significant indirect pathways existed between the nursing work environment and caring behavior. The mediation effect value of Path 2 (Nursing work environment → Empathy → Caring behavior) was 0.304, accounting for 35.3% of the total effect. Meanwhile, the mediation effect value of Path 3 (Nursing work environment → Psychological capital → Empathy → Caring behavior) was 0.318, accounting for 36.9% of the total effect. The chain mediating model is shown in Figure 2.

**Table 6:** Bootstrap analysis of the chain mediating model.

	Effect	Boot SE	Boot LLCI	Boot UCLI
Total effect	0.862	0.055	0.753	0.969
Direct effect	0.151	0.067	0.027	0.288
Total indirect effect	0.711	0.064	0.591	0.836
Path 1 (Nursing work environment → Psychological capital → Caring behavior)	0.089	0.058	−0.026	0.201
Path 2 (Nursing work environment → Empathy → Caring behavior)	0.304	0.064	0.184	0.433
Path 3 (Nursing work environment → Psychological capital → Empathy → Caring behavior)	0.318	0.058	0.215	0.444

## 4 Discussion

The total CBI score of hospice nurses was 125.5 ± 18.6 in this study, higher than that reported by Kaur et al. (2015). This may be due to the humanistic nursing care promotion by the government and healthcare industry in recent years. The “Healthy China 2030” plan calls for “strengthening humanistic care in medical services and enhancing nurses’ humanistic care capabilities” (Tan et al., 2019). Hospitals and scholars from various regions have actively responded to the national call. Furthermore, compared with nurses in regular departments, hospice nurses must allocate more effort to care for end-of-life patients. According to the questionnaire on caring behavior, the dimension of respect and connection scored the highest, followed by knowledge and skills, and the dimension of support and assurance scored the lowest. The literature suggests that hospice nurses lack the skills to provide personalized care to patients while performing work-related tasks. Therefore, nursing managers should combine humanistic care education with medical professional knowledge and skills to improve hospice care services.

The results indicate that the nursing work environment positively impacts the caring behavior of hospice nurses, which confirms Hypothesis 1. According to Takase et al. (2005), nursing is not a passive response to the environment, as each nurse possesses personal traits that interact with the working environment. In other words, a positive and supportive working environment, with adequate staff and nurse manager support, allows nurses to participate in hospital affairs, encourages good nursing service, and induces good nurse-physician relationships; thus, they feel an obligation to reciprocate favors received from the organization by providing high-quality care to patients (Liu and Aungsuroch, 2018; Cho et al., 2020). Additionally, previous studies suggest that a healthy work environment can reduce fatigue and maintain a higher level of job satisfaction, which leads to caring relationships (Shang et al., 2013). Hospice nurses face additional challenges, including caring for dying patients and providing bereavement support to their families. Therefore, hospital managers should reasonably allocate human resources, ensure that the rights and interests of hospice nurses are protected, and optimize the salary system to reduce their workload and psychological pressure while reflecting their support for nursing. Hospitals must provide a supportive nursing work environment to improve the caring behavior of hospice nurses. Table 7 shows how to improve the working environment from 5 dimensions at length.

**Table 7:** Recommended strategies to improve the working environment.

Dimensions	Interventions
Nurses participating in hospital affairs	(a) Establish a fully participative nursing management system to encourage their participation in organizational decision-making.
	(b) By building a platform for suggestions, managers encourage nurses to express themselves and communicate effectively.
	(c) Provide nurses the opportunities for career development and promotion, serving on hospital and nursing committees, participating in policy decisions, and involvement in the internal governance of the hospital.
Foundation of high-quality nursing services	(a) Managers should ensure continuing education programs for hospice nurses, so that nurses acquire updated knowledge and skills.
	(b) It is necessary to establish excellent hospice teams, including senior nurses and interdisciplinary healthcare professionals, so that team members have access to learn from each other and make progress.
	(c) Develop electronic registration system according to the specific situation of the department, so as to make scientific scheduling and provide nurses with flexible working hours.
Ability and leadership style of managers	(a) Hospital should train on transformational management, so as to cultivate head nurses with transformational leadership style and establish working atmosphere of respect and trust.
Sufficient human and material resources	(a) Nursing managers should formulate reasonable human resource allocation schemes, in order to make the ratio of nurses to hospital beds more reasonable.
	(b) Remote care has great advantages in the application of hospice patients, which can optimize the allocation of care resources.
Medical and nursing cooperation	(a) Establish effective communication mechanisms to promote medical and nursing cooperation.

According to the findings (Path 1), psychological capital had no significant mediating effect on the nursing work environment and caring behavior after controlling for age, health status, position, nursing title, marital status, and training experience. This does not support Hypothesis 2. Numerous studies indicate that psychological capital significantly differs by education and age (Babalola, 2009). Previous studies might focus on nursing students, whereas hospice nurses were selected as subjects in this study. The majority of hospice nurses have worked for at least three years. They have more clinical experience and higher psychological capital than nursing students. In summary, hospice nurses mobilize more positive psychological resources when they encounter difficulties at work. Moreover, most of the participants in this study had a bachelor’s degree or above. Due to their sense of superiority, individuals with higher levels of education were more likely to develop confidence and optimism. Finally, different evaluations may have contributed to different results.

After controlling for age, health status, position, nursing title, marital status, and training, this study indicated that empathy mediates the relationship between the nursing work environment and caring behavior (Path 2). The mediating effect accounted for 35.3%, which confirmed Hypothesis 3. As caregivers and companions of end-of-life patients, hospice nurses need empathy to communicate with patients, which helps improve their perception of their patients’ health and partly affects their hospice care quality (Shen et al., 2019). A comfortable nursing work environment can improve nurses’ empathy, affecting their caring behavior toward patients. Positive psychology methods have been widely applied in the nursing field. Cultivating nurses’ empathy is an active approach to preventing job burnout and compassion fatigue (Burtson and Stichler, 2010). Similarly, in the study of oncology nurses, it was found that improving empathy is an important strategy to reduce turnover intention (Wells-English, 2019). Nurses in good organizational environments have stronger empathy skills, can understand patients’ needs better, and communicate promptly, which reduces nurse–patient conflicts (Turner et al., 2019). Hospital managers must enhance empathy among hospice nurses to improve hospice care quality. This can be accomplished by understanding the needs of nurses and providing more opportunities for promotion and education.

Furthermore, the chain mediating the effect of psychological capital and empathy can be seen in the relationship between Chinese hospice nurses’ work environment and their caring behaviors (Path 3). The mediating effect accounted for 36.9%, which confirmed Hypothesis 4. This is the main theoretical contribution of this study. In other words, psychological capital and empathy can mediate the effect of the nursing work environment on the caring behavior of hospice nurses. A supportive work environment can increase internal psychological resources and capital (Xue et al., 2023). Individuals with high psychological capital also exhibit higher levels of self-efficacy and resilience. They believe they are more flexible to difficulties and complete clinical tasks more effectively. They also strive to achieve their goals and increase work engagement. According to studies, nurses involved in their work are more friendly and empathic toward their patients (Schaufeli, 2021). Nurses’ empathy plays an essential role in understanding the feelings and emotions of patients. Empathic hospice nurses are more likely to observe patients’ emotional changes and needs and to provide timely care in terms of physical, psychological, spiritual, and social needs to end-of-life patients, which also fits hospice connotations.

## 5 Conclusion

In conclusion, this study investigated how the work environment of Chinese hospice nurses affects their caring behavior. Specifically, we found a positive correlation between the nursing work environment and caring behavior. The mediating role of empathy in this relationship was verified. However, in this study, the mediating effect of psychological capital between the nursing work environment and caring behavior was insignificant. Their relationship needs further research in the future. Moreover, the results confirmed a chain mediation model between the nursing work environment, psychological capital, empathy, and caring behavior.

### 5.1 Limitations

There are several limitations to this study. First, this study used a cross-sectional design, making it impossible to determine causal relationships between variables. Second, the sample size was limited, which limits the generalizability of our findings. Hence, large-scale longitudinal studies for hospice nurses can be considered in the future. Third, the use of hospice nurses in certain regions of China as the research object in this study may have some impact on the external applicability of the result. Finally, the results were based on an online self-report survey, which is prone to inaccurate and biased responses from participants.

## Data availability statement

The original contributions presented in the study are included in the article/Supplementary material, further inquiries can be directed to the corresponding author.

## Ethics statement

The studies involving human participants were reviewed and approved by the Ethics Committee of Jiangsu Cancer Hospital. The studies were conducted in accordance with the local legislation and institutional requirements. Written informed consent for participation in this study was provided by the participants' legal guardians/next of kin.

## Author contributions

TW: Data curation, Investigation, Software, Writing – original draft. YL: Conceptualization, Writing – review & editing. JC: Methodology, Writing – review & editing. AM: Validation, Writing – review & editing. YW: Project administration, Writing – review & editing. LZ: Visualization, Writing – review & editing. BW: Funding acquisition, Writing – review & editing. BY: Formal analysis, Writing – review & editing. YZ: Funding acquisition, Resources, Writing – review & editing.
